# Explaining Differences in the Acceptability of 99DOTS, a Cell Phone–Based Strategy for Monitoring Adherence to Tuberculosis Medications: Qualitative Study of Patients and Health Care Providers

**DOI:** 10.2196/16634

**Published:** 2020-07-31

**Authors:** Beena E Thomas, J Vignesh Kumar, Chidiebere Onongaya, Spurthi N Bhatt, Amith Galivanche, Murugesan Periyasamy, M Chiranjeevi, Amit Subhash Khandewale, Geetha Ramachandran, Daksha Shah, Jessica E Haberer, Kenneth H Mayer, Ramnath Subbaraman

**Affiliations:** 1 Department of Social and Behavioural Research National Institute for Research in Tuberculosis Chennai India; 2 Department of Public Health and Community Medicine Tufts University School of Medicine Boston, MA United States; 3 Public Health Department Municipal Corporation of Greater Mumbai Mumbai India; 4 Center for Global Health Massachusetts General Hospital Boston, MA United States; 5 The Fenway Institute Fenway Health Boston, MA United States

**Keywords:** tuberculosis, medication adherence, mobile phone, mHealth, implementation science, qualitative research

## Abstract

**Background:**

99DOTS is a cell phone–based strategy for monitoring tuberculosis (TB) medication adherence that has been rolled out to more than 150,000 patients in India’s public health sector. A considerable proportion of patients stop using 99DOTS during therapy.

**Objective:**

This study aims to understand reasons for variability in the acceptance and use of 99DOTS by TB patients and health care providers (HCPs).

**Methods:**

We conducted qualitative interviews with individuals taking TB therapy in the government program in Chennai and Vellore (HIV-coinfected patients) and Mumbai (HIV-uninfected patients) across intensive and continuation treatment phases. We conducted interviews with HCPs who provide TB care, all of whom were involved in implementing 99DOTS. Interviews were transcribed, coded using a deductive approach, and analyzed with Dedoose 8.0.35 software (SocioCultural Research Consultants, LLC). The findings of the study were interpreted using the unified theory of acceptance and use of technology, which highlights 4 constructs associated with technology acceptance: performance expectancy, effort expectancy, social influences, and facilitating conditions.

**Results:**

We conducted 62 interviews with patients with TB, of whom 30 (48%) were HIV coinfected, and 31 interviews with HCPs. Acceptance of 99DOTS by patients was variable. Greater patient acceptance was related to perceptions of improved patient-HCP relationships from increased phone communication, TB pill-taking habit formation due to SMS text messaging reminders, and reduced need to visit health facilities (performance expectancy); improved family involvement in TB care (social influences); and from 99DOTS leading HCPs to engage positively in patients’ care through increased outreach (facilitating conditions). Lower patient acceptance was related to perceptions of reduced face-to-face contact with HCPs (performance expectancy); problems with cell phone access, literacy, cellular signal, or technology fatigue (effort expectancy); high TB- and HIV-related stigma within the family (social influences); and poor counseling in 99DOTS by HCPs or perceptions that HCPs were not acting upon adherence data (facilitating conditions). Acceptance of 99DOTS by HCPs was generally high and related to perceptions that the 99DOTS adherence dashboard and patient-related SMS text messaging alerts improve quality of care, the efficiency of care, and the patient-HCP relationship (performance expectancy); that the dashboard is easy to use (effort expectancy); and that 99DOTS leads to better coordination among HCPs (social influences). However, HCPs described suboptimal facilitating conditions, including inadequate training of HCPs in 99DOTS, unequal changes in workload, and shortages of 99DOTS medication envelopes.

**Conclusions:**

In India’s government TB program, 99DOTS had high acceptance by HCPs but variable acceptance by patients. Although some factors contributing to suboptimal patient acceptance are modifiable, other factors such as TB- and HIV-related stigma and poor cell phone accessibility, cellular signal, and literacy are more difficult to address. Screening for these barriers may facilitate targeting of 99DOTS to patients more likely to use this technology.

## Introduction

### Background

Tuberculosis (TB) is a leading infectious cause of mortality globally [1]. Poor adherence to medications may contribute to suboptimal TB care delivery outcomes by leading to higher mortality, treatment failure, posttreatment disease relapse, and development of drug-resistant TB strains [2,3]. In recent years, interest has increased in the use of digital adherence technologies (DATs), which include cell phone–based strategies, digital pillboxes, and ingestible sensors—to monitor and improve adherence to TB medications [4], especially given the growing concerns regarding more restrictive directly observed therapy (DOT) monitoring approaches [5-7]. Despite growing interest in DATs, data regarding these technologies’ acceptability, accuracy for measuring adherence, and effectiveness in improving treatment outcomes are lacking, especially in resource-constrained contexts [4,8].

99DOTS is a feature (ie, nonsmart) phone–based DAT that has been implemented widely in India’s National TB Elimination Program (NTEP) to monitor more than 150,000 patients taking therapy for drug-susceptible TB since 2015 [9]. The rapid expansion of 99DOTS in the NTEP was partly driven by the introduction in 2014 of daily TB medication dosing for all patients, replacing India’s prior intermittent (ie, thrice weekly) dosing regimen. Under India’s prior facility-based monitoring approach, most patients traveled to health centers for DOT. With the switch to daily dosing, program managers were concerned that traveling to health centers on a daily basis—rather than 3 times a week, as was previously the case—might be too challenging for patients [9]. In this context, remote monitoring of medication adherence using 99DOTS appeared to be a promising alternative [9].

Under the 99DOTS strategy, dispensing pills for each daily medication dose results in revealing a hidden phone number that patients call for free. A computer program records these phone call-reported doses, allowing remote visualization of each patient’s adherence record by health care providers (HCPs). 99DOTS’ potential advantages include its relatively low cost compared with other DATs, monitoring of individual adherence data in near real time, and automated notification of HCPs regarding potentially nonadherent patients, which has the potential to facilitate individualized care based on a patient’s risk for poor outcomes [9].

Despite these promising features, routine data and research studies have revealed suboptimal patient engagement with 99DOTS, that is, many patients do not call 99DOTS throughout the entire treatment course. For example, in a cohort study in Mumbai and Chennai conducted in parallel with this qualitative study, in which unannounced home visits were conducted on patients with TB across all months of therapy, only approximately two-thirds of patients had called 99DOTS in the 48 hours preceding the home visit [10]. Programmatic data from the rollout of 99DOTS to more than 20,000 patients in Mumbai similarly suggest that fewer than 40% of patients called 99DOTS to report at least 80% of their doses throughout the full treatment duration [9]. Suboptimal patient engagement may undermine 99DOTS’ benefits for measuring and promoting medication adherence and preventing loss to follow-up [10].

To our knowledge, only one study has been published evaluating the impact of 99DOTS on TB treatment outcomes. This study, conducted in four districts in Karnataka, compared TB treatment outcomes at HIV centers where 99DOTS was implemented with those at HIV centers where the technology was not rolled out [11]. Notably, implementation of 99DOTS at these centers occurred concurrently with the transition to daily (rather than thrice weekly) TB therapy. In addition, responsibility for dispensing TB medications and managing TB treatment was switched to HCPs at these HIV centers, whereas these tasks had previously been the responsibility of HCPs in the NTEP [11]. After adjusting for potential confounding factors, implementation of 99DOTS was significantly associated with 1.3 times increased adjusted relative risk of unfavorable outcomes, including death, lost to follow-up, and treatment failure [11]. Problems identified in that study, including increased workload for HIV center staff and reduced patient-provider interaction, have also been described in a study of 99DOTS conducted in Delhi [12]. In addition, most studies of other nonsmartphone-based technologies for promoting adherence to TB medications—in particular, one- and two-way SMS text messaging strategies—have not been shown to improve treatment completion [4,8]. These prior studies highlight the importance of understanding patient- and HCP-related barriers to the acceptance and the use of 99DOTS.

### Objectives

In this manuscript, we present findings from a qualitative study of patients and HCPs that aimed to understand the acceptability of 99DOTS in India’s NTEP. We interviewed drug-susceptible TB patients with and without HIV coinfection in three major Indian cities across both the intensive (early) and continuation (later) phases of therapy. Given the known problem of variable use of 99DOTS by TB patients, one goal of this analysis was to understand why some patients engage with 99DOTS, whereas others do not. In addition, we aimed to understand the acceptance of 99DOTS by HCPs with various roles in TB care provision, as the 99DOTS strategy represents a transformation in the approach by which HCPs monitor patients in the NTEP, which could have implications for work efficiency and quality of care. We analyzed patient and HCP findings using the unified theory of acceptance and use of technology (UTAUT), which synthesizes constructs that have previously been shown to predict the acceptance and use of novel technologies [13,14].

## Methods

### Ethics Approvals

The study protocol was approved by the Institutional Ethics Committee of the National Institute for Research in TB (NIRT; FWA00005104) on October 7, 2016; the Institutional Review Board of Brigham and Women’s Hospital (Partners Healthcare; FWA00000484) on January 31, 2017; and the Tufts Health Sciences Institutional Review Board (FWA00004517) on June 6, 2018. Written informed consent was obtained from all patients and HCPs who participated in the interviews.

### Study Setting

We recruited patients in Mumbai (population of about 18.4 million in the greater metropolitan area) and 2 cities in the south Indian state of Tamil Nadu—Chennai (population of approximately 7.1 million in the greater metropolitan area) and Vellore (population of about 480,000). All these cities have a relatively high TB burden in the general population [15,16].

### 99DOTS Implementation

With 99DOTS, medications are dispensed in blister packs wrapped in a custom envelope. 99DOTS envelopes were produced separately from the medication blister packs. As a result, TB personnel at the district or city level were responsible for wrapping the envelopes over medication blister packs before they were given to patients.

When a patient dispenses pills for a daily medication dose, this action breaks a perforated flap on the envelope, revealing a hidden phone number that the patient is requested to call for free. A computer program then records that the pill was *in-hand* and likely ingested. The phone number associated with each dose is unpredictable to the patient. When dispensing medications, pharmacists or other HCPs are responsible for explaining to patients how to call 99DOTS and its potential relevance for their care. Patients did not receive any monetary or nonmonetary incentives to call.

99DOTS allows HCPs to remotely *observe* a patient’s adherence history by logging in to a website on a computer or using an app on a mobile device, such as a smartphone. In this app, the 99DOTS dashboard presents a color-coded adherence calendar for each patient, with green showing that a call was made on a given day (suggesting that the dose may have been taken) or red showing that a call was not made (suggesting that the dose may not have been taken). These data are designed to help HCPs provide individualized feedback to patients regarding their adherence. A series of possible missed doses (ie, doses not *called in*) triggers automated SMS text message notifications to HCPs to help them identify patients who may be at higher risk for poor outcomes. HCPs are also provided with prioritized task lists recommending certain actions, such as calling to check-in with, or conducting a home visit for, a patient who has missed reporting a few doses in a row. As home visits are not normally conducted for patients taking unmonitored self-administered therapy, unless patients are lost to follow-up, we considered patient reports of multiple home visits by HCPs to be evidence that 99DOTS was potentially triggering such outreach.

Patient and HCP perceptions regarding whether 99DOTS facilitates more or less HCP-patient interaction were shaped by the alternative monitoring approach with which these individuals implicitly compared 99DOTS. For example, HCPs and some patients who had previously been treated for TB had experiences with facility-based DOT, which required patients to come to health facilities 3 times a week, therefore entailing a greater baseline level of HCP-patient interaction than 99DOTS. Other patients did not have experience with facility-based DOT and therefore implicitly compared 99DOTS with unmonitored self-administered therapy for other diseases, in which there is relatively minimal HCP-patient interaction apart from patient visits to health facilities for medication refills.

During the rollout of 99DOTS, in-person training took place at state-level offices, where nominated staff—from HIV treatment centers (commonly called antiretroviral therapy [ART] centers) or district TB programs—would gather at a single location. In addition, for ART centers, 2 virtual training sessions—each attended by more than 1000 HCPs—were conducted for individuals who could not attend centralized trainings. Subsequently, local trainings were conducted if requested by individual district TB programs. A team at Everwell Health Solutions, the organization managing 99DOTS’ implementation, monitored the rollout process to identify implementation gaps and provide monthly reports on patient engagement with 99DOTS. The HCPs reported problems via a WhatsApp group. These challenges were resolved remotely via WhatsApp or in person during regular field visits by personnel from Everwell Health Solutions.

HIV-coinfected patients underwent registration into 99DOTS and picked up TB medication refills at ART centers; however, home visits for these patients were conducted by HCPs in district TB programs. The National AIDS Control Organization (which manages ART centers) and the NTEP (which manages TB programs) were under different government bodies at the time of this study. As such, challenges in coordination between ART centers and district TB programs have the potential to impact linkage to care, and follow-up of HIV-coinfected patients started on TB therapy via 99DOTS.

### Recruitment of Study Participants and Collection of Qualitative Data

99DOTS was first rolled out to monitor HIV-coinfected patients diagnosed with TB at ART centers across India. In Chennai and Vellore, all TB patients in this study were HIV-coinfected individuals recruited from 5 different ART centers where 99DOTS had already been rolled out. Mumbai was the first city where 99DOTS was rolled out to facilitate monitoring of the broader (ie, mostly HIV uninfected) TB patient population under the care of the NTEP. We recruited mostly HIV-uninfected TB patients from 11 DOT centers in Mumbai. In India, approximately 95% of TB patients are HIV uninfected; however, given the unique challenges faced by HIV-coinfected patients, including having to concurrently take HIV and TB medications, and their generally poorer TB treatment outcomes [16], our goal in this study was to ensure that perspectives from HIV-coinfected and HIV-uninfected patients were well represented.

We had a goal of recruiting 60 TB patients using purposeful sampling, stratifying across the following criteria: (1) roughly equal representation of HIV-coinfected and uninfected patients; (2) roughly equal representation of patients in the intensive (early) and continuation (later) phases of therapy, as patients’ engagement with 99DOTS wanes throughout the course of TB therapy [9]; and (3) at least one-fifth of participants being women. Owing to recruitment in parallel across different study sites, we exceeded the target sample slightly, for both patient and HCP interviews, before this was recognized during routine biweekly virtual meetings among site personnel. Our anticipated sample size was somewhat large for a qualitative study, as we wanted to generate robust data for each of these stratified subgroups. One of the patient interviews was not transcribed and analyzed because the audio recording was muffled.

Patient interview guides included questions and follow-up probes to assess various aspects of patients’ experiences with 99DOTS, including counseling regarding this monitoring strategy, the process of calling 99DOTS, and HCP feedback and actions taken in response to patients’ adherence records. For HIV-coinfected patients, the interview guide included questions aimed at understanding the indirect impact of 99DOTS on HIV medication adherence. For each aspect of patients’ 99DOTS experience, questions assessed constructs for technology acceptance included in the UTAUT model.

We aimed to conduct interviews with 30 HCPs recruited using purposeful sampling, with the goal of including individuals with diverse roles in caring for HIV-coinfected and HIV-uninfected TB patients. We recruited HCPs who interact routinely with patients, including health visitors (individuals with at least a high school level of education who monitor TB therapy), senior treatment supervisors (individuals with at least a high school level of education who supervise health visitors), pharmacists, HIV counselors (who also counsel TB patients in HIV centers), TB counselors (who counsel TB patients in DOT centers), staff nurses, and medical officers (doctors with an MBBS or higher degree). We also recruited personnel with administrative roles, including data managers, TB officers (doctors who supervise TB care at multiple DOT centers), district TB officers (doctors who supervise TB care across a district), city TB officers (doctors who supervise TB care across a city), and district executive health officers (doctors who manage public health services across a district). In the representative quotations, we identify district TB officers, city TB officers, and district executive health officers as *higher-level administrative officers* to ensure that individuals are not identifiable.

We asked questions and follow-up probes to assess various aspects of HCPs’ experiences with 99DOTS, including using the 99DOTS dashboard to visualize patient data, notifications and task lists regarding high-risk patients and using these data to guide patient interactions. Questions for HCPs also assessed constructs in the UTAUT.

Interviews were conducted between February 2017 and August 2018. Interviews lasted 30 to 45 min and were conducted by 10 field researchers with a master’s degree in social work or another social science field. Field researchers at the Chennai, Vellore, and Mumbai sites underwent a uniform 2-day training at the NIRT in Chennai before starting data collection. All field researchers were taught to follow a uniform approach using a common interview guide. Patient interviews were conducted at DOT or ART centers where patients were receiving treatment. Patients were given nonmonetary compensation for their time equivalent to Indian rupees 100-150 (US $1.30-2.00), which consisted of helpful items such as dhal (lentils) or hygiene products.

HCP interviews were conducted in private spaces in DOT or ART centers or in other private locations of the study participant’s preference. Interviews were conducted in Tamil, Hindi, Marathi, or English using appropriately translated interview guides and were audio recorded, transcribed, and translated to produce English-language transcripts. Quantitative and qualitative data were deidentified before analysis, and care was taken to ensure that specific patients or health facilities cannot be identified based on the narrative excerpts included in the manuscript.

### Analytical Framework: UTAUT

The UTAUT integrates findings from prior models that were used to evaluate the acceptance and use of technologies [13,14]. By synthesizing diverse constructs used in prior models, the UTAUT identifies 4 broader constructs that explain technology acceptance: performance expectancy, effort expectancy, social influences, and facilitating conditions. Performance expectancy, or perceived usefulness, refers to the degree to which an individual believes that the technology will help with their medical care and daily life (for patients) or with work efficiency or the quality of care they are able to deliver (for HCPs). Effort expectancy, or ease of use, refers to how easy the technology is to use, for example, calling 99DOTS (for patients) or using the digital adherence dashboard (for HCPs). Social influences refer to the influence that other individuals—for example, family or community members (for patients) or other HCPs (for HCPs)—have on someone’s ability to accept or use the technology. Facilitating conditions refer to the quality of the organizational infrastructure that exists to support individuals using the technology; in this case, we assume that this refers to the training and infrastructure provided by the health system to ensure that 99DOTS functions appropriately for patients and HCPs.

In the UTAUT, 3 constructs—performance expectancy, effort expectancy, and social influences—influence behavioral intention to use a technology, which subsequently influences the actual use of a technology [13,14]. In contrast, facilitating conditions directly affect the actual use of a technology. Although all these constructs play a crucial role in shaping technology acceptance, in prior research, performance expectancy was found to be the strongest predictor of intention to use a technology [13,14].

### Analysis of Qualitative Data

We used a deductive approach for this thematic analysis, in which our coding was guided by the constructs in the UTAUT (Multimedia Appendices 1 and 2). An initial coding scheme was created based on collective discussion within the research team. Interview transcripts were independently coded by 3 researchers using Dedoose software (version 8.0.35; SocioCultural Research Consultants, LLC). Researchers met frequently to reconcile differences in code application and identify new themes emerging from the data. These new themes were incorporated into the coding scheme, and all transcripts were coded a second time using the revised coding scheme. After coding was complete, we identified key themes that could influence the acceptability and use of 99DOTS based on the general representativeness or salience of particular themes. We assembled these themes within the broader constructs of the UTAUT and identified representative quotations for each selected theme.

Although we could have quantified codes applied across interviews using Dedoose software, we did not do so for a few reasons. First, quantification implies that our findings are representative of those in a larger population; however, we used purposeful sampling of study participants. In addition, rather than just reporting common themes (ie, those reported most frequently), we also reported salient themes (ie, ones that seem important even if reported by a minority of participants). Second, quantification often implies that the same questions were asked to all participants in a systematic manner, as is done in structured surveys. Although field researchers underwent rigorous training and followed a uniform interview guide, the questions asked were open ended or semistructured, with a goal of eliciting narrative data that would be followed up with probe questions that could vary depending on the participant’s response. This malleable approach to interviewing is a strength of qualitative research, as it often yields unexpected findings; however, quantifying such findings could be misleading by implying a structured interviewing strategy.

In our analysis, we did not classify patients into those with *high acceptance* or *low acceptance* of 99DOTS. Although patients did have differences in the use of 99DOTS, individual patients often found different components of 99DOTS to be both acceptable and unacceptable depending on their individual circumstances. For example, the same patient might appreciate that 99DOTS averts the time spent traveling to health facilities (when hypothetically compared with facility-based DOT) but simultaneously find it difficult to use 99DOTS at home because of concerns about the disclosure of their TB diagnosis to family members. As such, we coded both positive and negative perceptions of 99DOTS that were reported by a given patient to provide a broader understanding of the factors that might increase or decrease the technology’s acceptability and use.

## Results

### Characteristics of the Study Participants

Excluding one nontranscribed interview, 62 patients with TB were interviewed, of whom 36 (58%) were men, 30 (48%) were HIV coinfected, and 34 (55%) were in the continuation phase of therapy. Patients’ ages ranged from 18 to 65 years (median 35 years). The median household monthly income was Indian rupees 8500 (IQR Indian rupees 7000-12,000), which is approximately US $111 (IQR US $92-157). With regard to educational attainment, 10 (16%) patients were illiterate, 21 (34%) had completed some schooling, 20 (32%) had a secondary school certificate, and 11 (18%) had pursued or completed postsecondary education (ie, college education or higher). With regard to occupation, 14 (23%) patients were unemployed, 13 (21%) were homemakers, 16 (26%) worked in the informal sector or as private low-wage employees (eg, waste collector or housekeeper), 18 (29%) worked in semiskilled or skilled trades or self-employment (eg, electrician or auto-rickshaw driver), and 1 (2%) worked in a high-wage profession (ie, engineer).

In total, 31 HCPs were interviewed, of whom 13 (42%) were men. HCPs’ ages ranged from 25 to 56 years (median 38 years). Of the 31 HCPs, 4 (13%) were health visitors, 4 (13%) were senior treatment supervisors, 2 (6%) were pharmacists, 2 (6%) were ART counselors, 3 (10%) TB were counselors, 1 (3%) was a nurse, 6 (19%) medical were officers, 2 (6%) were data managers, 2 (6%) were TB officers, 4 (13%) were district or city TB officers, and 1 (3%) was a district executive health officer.

### Findings From Patients With TB

**Figure 1:**
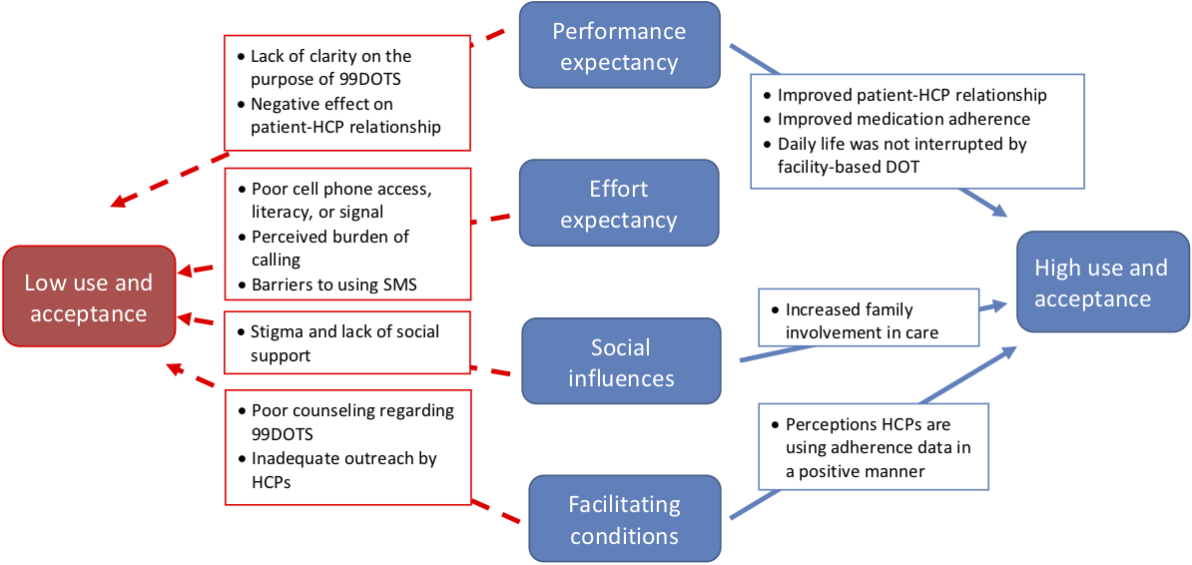
Key findings regarding the determinants of high and low acceptance and use of 99DOTS by patients with tuberculosis, based on the framework of the Unified Theory of Acceptance and Use of Technology. Note that there is no arrow between effort expectancy and high use and acceptance, because the findings did not reveal a meaningful relationship. DOT: directly observed therapy; HCP: health care provider; SMS: short messaging service.

Patient interviews revealed evidence of both high and low acceptability of 99DOTS (Figure 1).

### Determinants of High Patient Acceptability and Use of 99DOTS

With regard to performance expectancy (perceived usefulness), some TB patients perceived that 99DOTS contributed to improved patient-HCP relationships, improved medication adherence, and fewer interruptions to their daily life activities (Table 1 and Figure 1). Some patients reported that 99DOTS facilitated increased phone contact by HCPs. Such episodes of phone outreach as well as the perception that their adherence was being remotely observed facilitated a perception that the government was taking a personal interest in their health (Table 1, Q1) and was more *caring* about patients:

I am thankful that the government is taking good care of my health. It feels good that the government is calling me... they are caring.

50-year-old woman, HIV uninfected, continuation phase of therapy

Some patients also reported benefits of the SMS text messaging reminders in facilitating habit formation with regard to daily pill taking; they reported that these reminders “trained [their] minds” and created a new “awareness” about adherence (Q2 and Q3). Some HIV-coinfected patients reported that 99DOTS provided collateral benefit by serving as a reminder to take their antiretroviral medications:

It’s actually also reminding me to take ART tablets.

49-year-old man, HIV coinfected, intensive phase of therapy

A few patients who had also experienced the previous facility-based DOT model, which required thrice-weekly visits to health care facilities, reported benefits to their daily life, including saving money and time (Q4), from not having to visit health facilities as frequently in the 99DOTS model:

The previous approach [facility-based DOT] was time consuming and expensive too. This one [99DOTS] is good because you just have to take the pills and make the calls; it saves your time.

19-year-old woman, HIV uninfected, intensive phase of therapy

With regard to social influences, some patients said that 99DOTS increased social support by involving family members in their TB care (Q5); for example, family members routinely reminded or helped patients to call 99DOTS:

I have never forgotten [to call]. If I forget on one day, my daughter reminds me to make the call.

50-year-old woman, HIV uninfected, continuation phase of therapy

With regard to facilitating conditions, some patients noted that HCPs regularly used 99DOTS to guide their interactions, engaging in frequent phone outreach to patients or multiple home visits for counseling (Q6 and Q7). None of these patients reported having previous experiences with facility-based DOT. As such, their perception of increased HCP outreach may have been shaped by an implicit comparison of 99DOTS with unmonitored self-administered therapy, which is the standard of care for most other diseases. The perception that HCPs and the health system were using the adherence data from 99DOTS enhanced the motivation of these patients to continue using the technology. For example, the following patient appreciated how an HCP checked on his adherence and then wished him well:

One sir [HCP] called me and asked, “Are you taking tablets correctly?” I replied, “Sir I am taking them.” He would then say to me, “OK, take them regularly. Take care of your health. Be safe.”

35-year-old man, HIV coinfected, continuation phase of therapy

**Table 1:** Representative quotations on determinants of high patient acceptability and use of 99DOTS.

Determinant of acceptability		Quotations
**Performance expectancy**		
	99DOTS improved the patient–health care provider relationship	Q1. “[Being monitored by 99DOTS] doesn’t mean she [the health visitor] will forget about us; I feel that madam still remembers me... if I don’t call, she makes me understand what happens if I don’t take the pills.” (19-year-old woman, HIV uninfected, continuation phase of therapy)
	SMS text messaging alerts improved medication adherence	Q2. “Now I am remembering that I have to call at 11 O’clock. I have a new awareness that I should take tablets at 11’O clock.” (36-year-old man, HIV coinfected, continuation phase of therapy) Q3. “If we forget to call, the message alerts us to take tablets. So it trains our mind to take pills on time, so we will not forget to call. It is useful.” (52-year-old woman, HIV coinfected, continuation phase of therapy)
	Daily life was interrupted less when compared with facility-based directly observed therapy	Q4. “I prefer [99DOTS] because it saves our time. We can take the pills at home, and we can also do our domestic work.” (18-year-old woman, HIV uninfected, continuation phase of therapy)
**Social influences**		
	Increased family involvement in the patient’s care	Q5. “[My son] taught me, ‘You have to take 2 tablets per day and follow the arrow mark from the starting point’... He gave me my medicines from the beginning [of therapy] and reminds me to take tablets...he also dials the toll free number for me.” (43-year-old woman, HIV coinfected, intensive phase of therapy)
**Facilitating conditions**		
	Perception that health care providers are using the 99DOTS adherence data in a positive manner	Q6. “They have called me four or five times. They tell me that I missed taking my tablets and that I should call them. They will know [if] we don’t call them back.” (49-year-old man, HIV coinfected, intensive phase of therapy) Q7. Interviewer: “Did anybody come to your home from the hospital to see if you have taken your pills?”Respondent: “Yes... Madam came last week and another person also.” Interviewer: "How many times?”Respondent: “4 to 5 times.” (40-year-old man, HIV uninfected, continuation phase of therapy)

### Determinants of Low Patient Acceptability and the Use of 99DOTS

Determinants of low patient acceptability and the use of 99DOTS are presented in Table 2 and Figure 1. With regard to performance expectancy (perceived usefulness), many patients who were supposed to use the technology had trouble understanding what 99DOTS was until it was explained to them by the field researchers. This reflected a suboptimal understanding of the purpose of 99DOTS in their TB care. Some patients also felt that 99DOTS resulted in a poorer patient-HCP relationship by reducing the frequency of face-to-face contact. This led to some patients feeling *isolated* (Table 2, Q8), whereas others described difficulties in reporting potential adverse reactions to medications because of the reduced frequency of visits:

I developed leg numbness during treatment... Maybe if I came to the center [more frequently] and discussed my problem with the doctor they could have given some suggestion. But I am able to share my problems only once a month with the doctor.

32-year-old woman, HIV coinfected, continuation phase of therapy

Other patients did not appreciate receiving negative feedback from HCPs for not calling 99DOTS (Q9), although they reported regularly taking their pills:

The HCP scolded me for not calling... If the patient doesn’t give the missed call, then the government thinks the patient has not taken the tablet.

65-year-old woman, HIV coinfected, continuation phase of therapy

Several concerns emerged regarding effort expectancy (ease of use). A few patients reported not having any access to a phone either because they live in situations (eg, college dormitory with restrictive policies) in which they were not allowed to keep or use cell phones (Q10) or because they and their family members do not own a phone:

I live in an interior village. No one has a phone I can use. Only my sister has a phone. I don’t have a phone in my own hand. We don’t have many resources, Madam.

32-year-old man, HIV coinfected, continuation phase of therapy

A more prominent problem was shared cell phone use (Q11 and Q12), which reflected problems with accessibility (eg, another family member keeps the cell phone), literacy (eg, older patients did not know how to use the phone), or both, as described in the following quotation:

I don’t know how to use it, so I do not keep the phone with me... My son leaves our hometown for employment. I can give the missed calls when he is at home... what can I do if my son is not available?

65-year-old woman, HIV coinfected, continuation phase of therapy

Sharing of cell phones often resulted in patients using 99DOTS in ways that would result in adherence data not being reported or being reported inaccurately in the 99DOTS system. For example, patients reported medication doses using a third-party phone number not registered with 99DOTS (Q12), called the same phone number over several days (Q13), or called all the phone numbers listed on an envelope on the same day (Q14). In addition, patients reported separating envelopes from medication blister packs to have family members call 99DOTS on their behalf, which could result in calls into the 99DOTS system happening independent of actual pill ingestion:

I leave that cover [envelope sleeve] with my son and just take the tablets with me [to consume at work]... I ask him at night [if he made the call].

50-year-old woman, HIV coinfected, continuation phase of therapy

Patients reported other technical barriers to using 99DOTS, including getting a busy signal when calling the 99DOTS number (Q15), lack of electricity in the home to keep their cell phones charged (Q16), or inability to read the number on the 99DOTS envelope (Q17). The most common technical problems related to challenges with SIM cards (eg, lost or nonfunctional cards, Q11 and Q12) or patients lacking cellular signal in their homes (Q18):

There is not enough [cellular] signal in the area where I live, so I borrow a phone from someone else and call the toll-free number.

55-year-old man, HIV coinfected, continuation phase of therapy

A few patients also described a sense of *fatigue* with calling 99DOTS, especially in the continuation phase of therapy (Q19). The following patient describes this feeling of decreasing motivation as treatment progressed:

Yes, my interest in calling has decreased compared to the initial phase... Now I don’t [call after taking medication].

35-year-old man, HIV uninfected, intensive phase of therapy

Other patients articulated a vaguer feeling of tiredness or sleepiness after taking medication that prevented them from calling (Q20).

Several patients also reported barriers in ease of use for SMS text messaging reminders, including language barriers (SMS text messages were often sent in English rather than local Indian languages, Q21) and not noticing 99DOTS SMS text messaging reminders (Q22), in light of the high volume of *spam* SMS text messages sent by advertisers.

With regard to social influences, TB- and HIV-related stigma were major barriers to using 99DOTS. Some patients had not revealed their TB or HIV diagnoses to their family members because of stigma and, as a result, wanted to take their pills in the dark (when family members were sleeping) or in a private place outside the home, for fear of these diagnoses becoming disclosed (Q23 and Q24). One patient described having to step away from others to make the 99DOTS phone calls:

I am very worried about my children coming to know [about the TB diagnosis]... so I am making calls while hiding from others.

51-year-old man, HIV coinfected, intensive phase of therapy

The 99DOTS envelope served as a barrier to being able to cut up medication blister packs to facilitate taking TB medications discreetly. Patients also had concerns when HCPs visited their homes—in response to a lack of phone calls registered in the 99DOTS dashboard, as these visits sometimes resulted in disclosure of the patient’s TB and/or HIV diagnoses to family members (Q25).

In terms of facilitating conditions, many patients reported poor counseling by HCPs regarding how to use 99DOTS and what its potential benefits might be (Q26 and Q27). For example, one patient did not realize that the SMS text messaging reminders he was receiving were from 99DOTS; he had assumed that they were from his son’s employer:

I didn’t know why [the SMS reminders from 99DOTS] came... I thought that I was getting messages from construction sites that my son works at.

55-year-old man, HIV coinfected, continuation phase of therapy

Some patients expressed disappointment in the lack of outreach by HCPs via phone calls or home visits. The reasons that patients felt disappointed due to lack of outreach varied. A few patients had prior experiences with facility-based DOT and implicitly compared this prior model with 99DOTS, which involves a lower frequency of HCP-patient interaction. Other patients felt disappointed because they received no outreach at all from HCPs by phone or home visit (Q28) or because HCPs had called to say they would conduct a home visit but did not actually do so, as described in the following quotation:

[HCPs] told me that they will come to my home, but they never came.

28-year-old man, HIV uninfected, continuation phase of therapy

**Table 2:** Representative quotations on determinants of low patient acceptability and use of 99DOTS.

Determinant of low acceptability		Quotations
**Performance expectancy**		
	Technology negatively affects patient-provider relationship	Q8*.* “One person came to see me since I started regularly taking TB tablets. They came and saw me once and that was it... I told them no one is visiting me and that I feel isolated. But nobody is interested in my worries.” (32-year-old man, HIV coinfected, continuation phase of therapy) Q9. “We wake up at 4 in the morning and we are fasting all day [for Ramadan], so in the morning there is no time to call... madam told me that I had not called and scolded me.” (50-year-old woman, HIV uninfected, continuation phase)
**Effort expectancy**		
	Inability to call because of lack of phone access or restricted phone access	Q10. “I have a phone in my office. But students are not allowed to use mobile phones until we go home.” (19-year-old man, HIV coinfected, continuation phase of therapy)
	Inability to call because of shared phone use	Q11. Interviewer: “Has it ever happened that you had not made the call because of your phone problems?” Respondent: “[When f]ather is not at home whole day or when there is a SIM card network issue also.” (20-year-old woman, HIV uninfected, continuation phase of therapy) Q12. “My mother used to take [the cellphone] with her... for those days I couldn’t call... There was an alternative phone — my brother-in-law’s mobile phone. After taking medication I would tell my brother-in-law... But he ended up losing his SIM card.” (34-year-old man, HIV coinfected, intensive phase of therapy)
	Inappropriate calling of phone numbers in the envelopes (may lead to inaccurate adherence information)	Q13. “I saved the toll free number in the first blister [of the envelope] and called that number only. Later they said I should not call like that. They advised to call according to [the corresponding] blister [for each day].” (50-year-old man, HIV coinfected, continuation phase of therapy) Q14. “My daughter calls...but she does not call daily. She calls all the numbers on the strip at once after all the medication [in the blister pack] has been taken.” (55-year-old man, HIV coinfected, continuation phase of therapy)
	Other barriers to cell phone use or calling 99DOTS	Q15. “The first time I call, it gives a busy signal . . . after thinking that I dialed a wrong number, I dial the number again and it works... this has happened two or three times.” (43 year-old-woman, HIV coinfected, intensive phase of therapy) Q16. “I am staying in a hut, so I don’t have electricity in my home; we burn [wood] sticks to get light.” (49-year-old man, HIV coinfected, intensive phase of therapy) Q17. “My vision is not clear enough to see the small print [on the envelope]... I get help from my daughter or someone who can see the letters and call.” (42-year-old man, HIV coinfected, continuation phase of therapy) Q18. “Yes, sometimes there are network [cellular signal] issues at my house*.”* (25-year-old woman, HIV uninfected, continuation phase of therapy)
	Perceived high burden of calling or “technology fatigue”	Q19. “[I am] tired of calling daily.” (19-year-old man, HIV uninfected, continuation phase of therapy) Q20. “I forget to call Madam... I fall asleep as soon as I take [the medication].” (42-year-old man, HIV coinfected, continuation phase of therapy)
	Barriers to using SMS text messaging reminders	Q21. “If I receive a message in Tamil, I’ll try to read by spelling out the letters... but I don’t know how to read in English.” (30-year-old man, HIV coinfected, continuation phase of therapy) Q22. “Honestly I did not notice [the SMS reminders] or I did not check my phone for messages.” (25-year-old woman, HIV uninfected, continuation phase of therapy)
**Social influences**		
	Stigma and lack of social support present barriers to patient engagement	Q23. “We do not take medication in front of others. People think TB is the worst disease and spreads by touching and that even if you talk, the disease will spread... all people think I am dirty; nobody wants to come close to me, so that’s why we take our pills behind closed doors.” (19-year-old woman, HIV uninfected, continuation phase of therapy) Q24. “Sometimes I take [medication] when nobody else is at home or... when everyone at home is sleeping... Sometimes I leave my home to take the tablets.” (19-year-old man, HIV coinfected, continuation phase of therapy) Q25. “[The HCPs visiting my house] revealed my status. They told others that I have TB... I feel unworthy to live after others came to know that I have AIDS.” (54-year-old man, HIV coinfected, intensive phase of therapy)
**Facilitating conditions**		
	Poor counseling regarding 99DOTS	Q26. “One informational paper was given but... I thought it was useless and threw it out. Then when I came again to collect medicine, they asked me why I was not calling and I told them that I never received a number to call.” (27-year-old woman, HIV uninfected, intensive phase of therapy) Q27. “Sometimes I wonder, why do they ask us to call? What is the reason for calling?” (29-year-old man, HIV coinfected, intensive phase of therapy)
	Perceptions of inadequate or negative outreach by health care professionals	Q28. “They [HCPs] didn’t call... no one visited my house yet either.” (35-year-old man, HIV coinfected, intensive phase of therapy)

### Findings From HCPs

**Figure 2:**
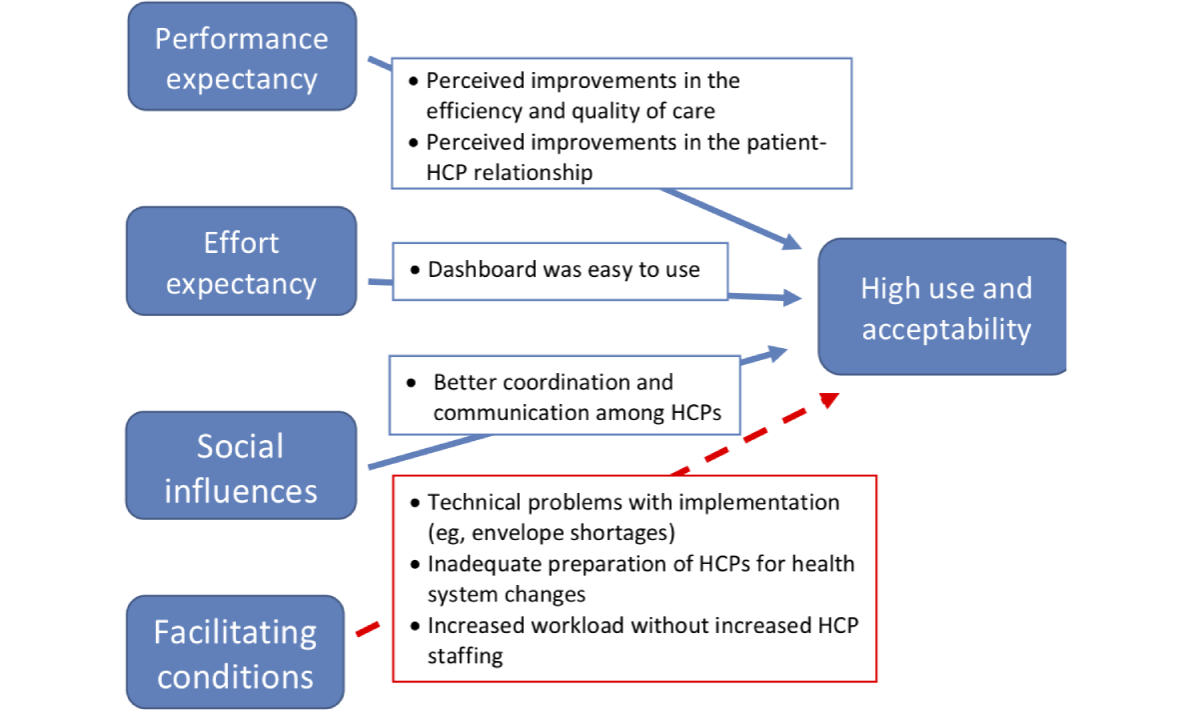
Key findings regarding the determinants of acceptance and use of 99DOTS by health care providers, based on the framework of the Unified Theory of Acceptance and Use of Technology. Most determinants suggest high acceptance, except for facilitating conditions, as indicated by the red dotted line, which indicates a negative association. HCP: health care provider.

The HCP interview findings generally reflected high acceptance of 99DOTS, with a few exceptions (Figure 2 and Table 3). With regard to performance expectancy (perceived usefulness), HCPs generally found 99DOTS to be a helpful tool for monitoring patients and felt that the technology improved work efficiency. Higher-level managers found the ability to view patient adherence and outcomes at an aggregate level to be helpful, as described in the following quotation:

I am able to see the adherence of the patient in an analytical way, so it helps me and my staff to directly monitor the patient. I can randomly call patients from Mumbai. It’s very easy. Without it, I would have to open my Excel [spreadsheet] to see [patient details].

A higher-level administrative officer from Mumbai

HCPs who interacted directly with patients found that automated notifications helped them to prioritize high-risk patients and encourage phone outreach (Table 3, Q29), which strengthened the patient-HCP relationship and resulted in early identification of medication adverse effects:

There was one patient who was registered for 99DOTS, but she was not calling. When she was contacted... she said she was experiencing some side effects of the drug... So definitely it is helpful.

A higher-level administrative officer from Mumbai

Some HCPs reported feeling that they had more time to perform other tasks (such as documentation), given the reduced time spent on directly observing medication ingestion by patients (Q30). HCPs also perceived that patients had improved adherence because of the feeling that they were being *cared for* by the HCPs on the other side of the monitoring technology (Q31). They also felt that 99DOTS enhanced the frequency and ease of outreach to patients by phone:

Actually, due to the 99DOTS program, communication [with the patient] has increased... [99DOTS] is useful...we can call the patient and inform them to take pills, which is beneficial.

A TB Officer from Mumbai

However, in contrast, a few HCPs described concerns about the accuracy of the adherence record and the quality of the patient-HCP interaction in the absence of direct observation, as noted in the following excerpt:

[Patients] may call us even without taking the tablet too. We cannot directly observe them with 99DOTS... [patients] may dial the phone number for our sake and throw out the tablets.

TB counselor in Tamil Nadu

With regard to effort expectancy (ease of use), HCPs generally found the 99DOTS adherence dashboard to be easy to use and preferred electronic data entry into the 99DOTS dashboard, as compared with written records (Q32). They also found it easy to generate reports through the 99DOTS dashboard in a manner that prioritizes high-risk patients, as described in the following quotation:

It’s easy when it comes to reports. Immediately the report can be pulled out to determine which patients need to be given priority.

A medical officer from Tamil Nadu

The HCPs perceived that some patients found 99DOTS to be easy to use, but HCPs also affirmed findings from the patient interviews that many patients, especially those with low literacy or educational level (Q33), had challenges using 99DOTS:

[Patients] find it easy, but it goes hand in hand with literacy rate.

A higher-level administrative officer from Mumbai

With regard to social influences, HCPs described high uptake and utilization of 99DOTS by their supervisors and peers, such that 99DOTS improved coordination among HCPs, which likely served as a social influence that increased its acceptability (Q34). For example, the following nurse said that she is easily able to contact her colleagues in a timely manner due in part to 99DOTS:

We [HCPs] always have contact [with each other] through mobile phones. After registering the patient [in 99DOTS] they will get a message, and then they will call us and ask for a report on the patient’s status and we will inform them. I have everyone’s mobile number.

A staff nurse from Tamil Nadu

Although HCPs described positive findings across most UTAUT constructs, many HCPs described suboptimal facilitating conditions during the rollout of 99DOTS. Technical problems with implementation included stock outs of 99DOTS envelopes for wrapping medication blister packs (Q35) and the dashboard not being updated with adherence information, despite patients calling to report doses (Q36). Some HCPs reported a lack of training in 99DOTS (Q37) and difficulty in understanding how to practically apply the training (Q38) due to lack of demonstrative teaching:

Program clarity was there, but how it was going to be implemented in the field was not that clear, because there was no live demonstration of patient [interaction] and enrollment during the training session.

A higher-level administrative officer from Mumbai

Some HCPs also described inadequate preparation of personnel for 99DOTS implementation and unequal changes in workload, without commensurate increases in personnel. For example, HCPs in ART centers were previously not responsible for dispensing TB medications, as HIV-coinfected patients diagnosed with TB had previously been referred to DOT centers close to their homes to start treatment (Q39). However, with implementation of 99DOTS, because HCPs in DOT centers were no longer monitoring TB patients using facility-based DOT, the responsibility for dispensing TB medications was transferred to pharmacists at ART centers, as these pharmacists also dispensed ART to these patients for their HIV. As a result, medical officers (ie, doctors), pharmacists, and counselors at ART centers found that their workload increased substantially (Q39), as they had new responsibility for monitoring and counseling patients regarding their TB treatment and use of 99DOTS, without increased personnel support:

For just the ART tablets, we would count out missing pills and document them. Now for TB tablets, we also have to note down how many pills are missing... We have to fill so many pages... So workload is difficult. We did not have staff allocated for that change. We just have to do all the work.

A counselor from Tamil Nadu

These concerns by staff at ART centers were in contrast to the more general opinion among TB staff, in centers that did not take care of HIV-coinfected patients, that 99DOTS decreased workload.

**Table 3:** Representative quotations on determinants of health care provider acceptability and use of 99DOTS.

Determinant of health care provider acceptability		Quotations
**Performance expectancy**		
	Perceived improvements in the efficiency and quality of care	Q29. “The health visitors and TB officers were coming to know who had missed doses in a span of one or two successive days. So it became easy for the staff to contact those patients and come to know the reason why patients were missing doses or why they had not called.” (A higher-level administrative officer from Mumbai) Q30. “Because [99DOTS uses remote monitoring] instead of daily supervision [i.e., DOT], I can relax and focus on my work... if I am only meeting a patient once a month, it reduces my work load.” (A health visitor from Mumbai)
	Perceived improvements in the patient-HCP^a^ relationship	Q31. “The patient mindset has changed...the patient starts to feel that [HCPs] are taking care of me; that is why they are telling me to make a phone call. So he will continue to take tablets correctly.” (A counselor from Tamil Nadu)
**Effort expectancy**		
	Dashboard was easy to use	Q32. “[99DOTS] is easy from all angles. We are entering data manually as well as updating everything on the online portal. [99DOTS] is better to handle compared to manual entry for maintaining the [patient] register.” (A senior treatment supervisor from Tamil Nadu)
	Patient ease of use was perceived to be variable, depending on educational level	Q33. “Generally if the patient completed a 10th grade education they know how to use the 99DOTS program.” (A senior treatment supervisor from Mumbai)
**Social influences**		
	Better coordination and communication among HCPs	Q34. “High and medium priority patients are flagged... The patient list is taken and given to the counselor. A message is forwarded to the [senior treatment supervisors]. Every Thursday, a HIV/TB meeting is conducted by the HIV/TB coordinator. Data managers share patient information with the medical officers...” (A medical officer from Tamil Nadu)
**Facilitating conditions**		
	Technical problems with implementation	Q35. “One of the main problems with 99DOTS is that there was shortage [of envelopes].” (A medical officer from Tamil Nadu) Q36. “I have a few patients call repeatedly...the number is correct and registered in the 99DOTS system. They have been getting the ‘thank you’ message also [which confirms the call was received]. But his treatment status does not get updated on the dashboard.” (A higher-level administrative officer from Mumbai)
	Inadequate training of HCPs for health system changes	Q37. Interviewer: “Now, before implementation of 99DOTS program did you receive any training?” Respondent: *“*No, nowhere.” (A health visitor from Mumbai) Q38. “Initially when they were training [staff in 99DOTS], I did not understand anything. Then I took the class again. That time we got a better understanding of the project. I could only get a better understanding after I had seen patients individually.” (A pharmacist from Tamil Nadu)
	Changes in workload with 99DOTS implementation without changes in HCP staffing	Q39. “Compared to ART [HIV treatment] this is difficult to handle... We didn’t have any connection with DOTS [the national TB program] before. Once we diagnosed TB, we would give a referral form, and patients would get tablets in the DOT centers... Compared to our previous work, ART staff are doing much more work now.” (A medical officer from Tamil Nadu)

^a^HCP: health care provider.

## Discussion

### Implications of Findings From Patients With TB

In this multisite study of the large-scale rollout of a novel DAT, we identified multiple factors that help to explain differences in the acceptability of 99DOTS as a monitoring strategy for TB patients and HCPs. TB patients often had substantially different perspectives regarding the technology, which were shaped by variability in patients’ access to resources and social circumstances, including levels of TB- and HIV-related stigma in their families and communities.

With regard to resources, lack of reliable cell phone access—including not having a phone, only having access to a phone shared with family members, lack of electricity for phone charging, and unreliable cellular signal—considerably hindered ease of use (effort expectancy). However, cell phone–related barriers went beyond access alone. Some patients who had reliable cell phone access reported technology fatigue, expressed as *loss of interest* in calling 99DOTS. Technology fatigue has also been reported in previous studies of nonsmartphone-based TB treatment monitoring strategies, in particular two-way SMS text messaging approaches [17]. This finding may be particularly concerning because prior evidence suggests that performance expectancy, or perceived usefulness of the technology, is the strongest predictor of intention to use a technology. Consistent with these findings, SMS text messaging reminders alone or two-way SMS text message–based strategies (ie, in which patients send SMS text messages to report doses taken) have been found to be ineffective or less effective than strategies that combine SMS text messages with other interventions to promote TB and HIV medication adherence [17-22]. These findings suggest that reminders and remote monitoring may be insufficient and that strategies that use technology to facilitate human interaction, rather than replacing it, may be more effective in improving medication adherence, a principle supported by recent trials of DATs in TB and HIV care that have had more successful outcomes [23-25].

Patients’ individual social circumstances also shaped their acceptance of 99DOTS. For example, some patients felt that 99DOTS improved the patient-HCP relationship by facilitating more frequent phone outreach from HCPs, whereas other patients felt isolated by the reduced face-to-face contact with HCPs under 99DOTS. In addition, some patients, who presumably had low TB- and HIV-related stigma within their household, felt 99DOTS involved family members in their TB care in a beneficial manner. In contrast, other patients were so concerned about TB- and HIV-related stigma that they hid their disease (and pill taking) from family members, which made it challenging to regularly call 99DOTS.

For patients concerned about stigma, alternative monitoring strategies may be more or less conspicuous to family members and others in the community, depending on individual circumstances. For example, facility-based DOT, in which patients visit local health centers, where HCPs observe them take their medications, was previously widely used for monitoring in India’s TB program. In this approach, individuals who work outside of the home may be able to visit health facilities during working hours without their family members’ knowledge; however, for individuals who spend most of their time at home, engagement with facility-based DOT may be more noticeable than taking and reporting medications via the 99DOTS strategy.

In contrast, our findings suggest that unmonitored self-administered therapy—in which patients take their medication blister packs home with them without having to call 99DOTS—is a strategy that would be less conspicuous for most patients with concerns about TB-related stigma. Such an approach makes it easier to take medications discreetly for a few reasons. First, when medication blister packs were not wrapped in 99DOTS envelopes, patients could cut up blister packs into smaller strips that allowed them to be hidden more easily within the home. Second, cutting up blister packs into small strips also allowed patients to easily carry these medications in their pockets, so that medications could be ingested discreetly outside the home (eg, in a local temple in one case). Third, not having to dispense and call the 99DOTS phone number considerably reduced the visibility of the act of pill taking, particularly for patients who might rely on a family member’s cell phone to call 99DOTS. For example, one patient described not being able to call 99DOTS because she took her medications discreetly in the dark after other family members went to sleep.

These various factors help to explain suboptimal patient use of 99DOTS [9,10], which contributes to its suboptimal accuracy for measuring adherence [10]. In our concurrent patient cohort study assessing 99DOTS’ accuracy for measuring adherence [10], approximately one-third of patients had not called 99DOTS in the 3 days before our study team’s unannounced home visit. However, more than three-fourths of these patients were actually taking their TB medications, as measured by a positive urine isoniazid test. Such patterns make sense in light of our qualitative findings, as many patients had barriers to calling 99DOTS (eg, stigma and lack of phone access) but still expressed motivation to take TB medications. That cohort study also found that a small proportion of patients shown as being *adherent* on the dashboard were calling 99DOTS but not actually taking their TB medications [10]. The qualitative findings in this study suggest that some of these inaccurate signals may be attributable to shared cell phone use, which sometimes inadvertently led to inappropriate calling of 99DOTS to report doses, for instance, when envelopes were separated from blister packs and given to family members to call in doses.

Our findings also have implications for the potential strengths and weaknesses of using other DATs to monitor TB medication adherence in this context. For example, some of the barriers to ease of use with 99DOTS could potentially be rectified by providing TB patients with alternative DATs that have a lower patient burden. For example, digital pillboxes record openings and closings of the pillbox as a proxy for medication ingestion. As patients have to open these pillboxes to access their medications, the patient burden is often considered to be lower than two-way SMS text messages or cell phone–based DATs such as 99DOTS, which require patients to take an additional step (SMS text message or phone call) to report a dose [4]. As such, the use of digital pillboxes or other alternative technologies may help to expand the reach of DATs to TB patients who have unreliable cell phone access or who lose interest in calling every day.

In contrast, patients who face high TB- and HIV-associated stigma in their family environment may have challenges not only with 99DOTS but also with alternative DATs, such as digital pillboxes or video DOT, because all these technologies result in pill taking being more noticeable to family members than might be the case with unmonitored self-administered therapy. For example, a digital pillbox would likely be even more difficult to hide from family members than the 99DOTS medication envelopes and, therefore, may have lower acceptability for some patients. As such, it is critical that rigorous research involving diverse patient populations be conducted before novel DATs are rolled out in the Indian setting to better understand their potential strengths and weaknesses in this context.

### Implications of Findings From HCPs

The acceptability of 99DOTS for HCPs was generally high. HCPs with a variety of roles in the NTEP found the 99DOTS dashboard to be intuitive and user friendly and most had a general perception that the system had improved efficiency and quality of care, the patient-HCP relationship, and coordination among different HCPs involved in providing care.

However, some HCPs raised concerns about the facilitating conditions for the 99DOTS rollout. In particular, for HCPs at ART centers, the implementation of 99DOTS coincided with a *single window* approach to dispensing and monitoring both HIV and TB medications for HIV coinfected TB patients, partly because 99DOTS allowed centralized monitoring of TB medication adherence. As a result, counselors, pharmacists, and medical officers at ART centers voiced concerns about the increased workload of having to provide longitudinal TB care for the first time, in addition to their usual responsibilities. A recent study conducted at ART centers in Karnataka identified similar problems and found that implementation of 99DOTS with a *single window* approach may have adversely affected TB patient outcomes [11]. These concerns highlight the importance of anticipating changes in workload with the implementation of DATs. It was notable that despite the concerns raised by some HCPs regarding facilitating conditions, most HCPs still seemed to appreciate many features of 99DOTS.

We have previously described how DATs have the potential to transform the delivery of TB care if implemented in a manner that is patient centered [4,26]. For example, DATs have the potential to help HCPs to remotely view adherence data, prioritize their outreach efforts to focus on patients at highest risk, and provide differentiated care to patients based on their individual adherence records. Our findings suggest that HCPs understood the potential benefits of these innovations and were generally open to them. However, a limitation of this qualitative study is that it only assessed HCPs’ perceptions of 99DOTS’ benefits, rather than the actual accuracy of this technology or its impact on patient outcomes. 99DOTS’ acceptability may have been overestimated because of social desirability bias (ie, patients or HCPs telling interviewers what they thought they wanted to hear). As already noted, a cohort study, conducted concordantly with this qualitative study, has revealed concerning limitations in 99DOTS’ accuracy for measuring true TB medication adherence [10]. Although HCPs may be enthusiastic about 99DOTS, there may be serious shortcomings in the quality of the adherence data they are receiving, which is particularly concerning when these data are used for public health and clinical decision making.

Another limitation of our study is that we used a deductive approach to analyzing data based on the fact that the constructs in the UTAUT framework have previously been shown to predict technology acceptance and use. A limitation of this analytical approach is that we could have potentially missed some findings from these interviews that did not clearly fit into this predetermined framework.

As our patient sample only included individuals currently being monitored by 99DOTS, we were not able to compare patients’ perceptions of 99DOTS with alternative care delivery strategies, such as facility-based DOT or unmonitored self-administered therapy. Although a few patients in our sample compared their experiences with 99DOTS with prior experiences with facility-based DOT, a larger sample would be required to draw definitive conclusions about whether and what types of patients prefer one monitoring approach over another. In addition, patients in our sample with prior experience of facility-based DOT voiced preference for 99DOTS primarily because they did not have to travel to health facilities on a regular basis; however, the same benefit could be achieved with unmonitored self-administered therapy. Future qualitative research should investigate patient preferences for different types of treatment monitoring strategies.

Future research may also evaluate whether the provision of monetary or nonmonetary incentives—or alternatively, disincentives, such as transition to facility-based DOT for patients who do not call 99DOTS—could facilitate greater engagement with the technology. Such studies incentivizing patients to call 99DOTS should be conducted with caution. Existing research comparing 99DOTS’ accuracy against adherence measured via urine isoniazid testing suggests that a considerable proportion of nonadherent patients, as confirmed by negative urine isoniazid test results, still call 99DOTS to report taking medications [10]. The provision of incentives to increase patient engagement with 99DOTS has the potential to worsen this problem, which would further decrease the capacity of 99DOTS to identify patients who are truly not taking their TB medications.

### Conclusions

In this multisite study of the large-scale implementation of 99DOTS, we found that 99DOTS had high acceptability among HCPs, but patients in India’s public sector TB program had variable, sometimes negative, perceptions. Our findings highlight some barriers to the acceptance and use of 99DOTS that could potentially be mitigated through better rollout and implementation of the technology, including improvements in 99DOTS counseling (for patients) or training (for HCPs) and better anticipation of changes in workload for HCPs. In addition, screening TB patients for specific barriers to accepting or using 99DOTS, such as high levels of TB- and HIV-associated stigma in the household, lack of reliable cell phone access, or lack of cell phone literacy—could help target 99DOTS to patients who are more likely to engage with this technology while also identifying patients for whom alternative monitoring approaches (eg, digital pillboxes, video DOT, in-person DOT, or unmonitored self-administered therapy) should be offered.

## Appendix

Multimedia Appendix 1 Coding Scheme for the 99DOTS patient interviews.

Multimedia Appendix 2 Coding scheme for the 99DOTS HCP interviews.

## Abbreviations

ART: antiretroviral therapy
DAT: digital adherence technology
DOT: directly observed therapy
HCP: health care provider
NIRT: National Institute for Research in TB
NTEP: National Tuberculosis Elimination Program
TB: tuberculosis
UTAUT: unified theory of acceptance and use of technology
