# Supplementary material for: Explaining Differences in the Acceptability of 99DOTS, a Cell Phone–Based Strategy for Monitoring Adherence to Tuberculosis Medications: Qualitative Study of Patients and Health Care Providers
Source: JMIR Mhealth Uhealth. 2020 Jul 31;8(7):e16634. doi: 10.2196/16634 (PMC7428900; doi:10.2196/16634)
Supplement: Multimedia Appendix 1 [file mhealth_v8i7e16634_app1.pdf]

Figure S1. Coding scheme for 99DOTS patient interviews

Component of the UTAUT framework	Parent nodes	Child nodes
**Performance expectancy**	Patient understanding of 99DOTS	Lack of clarity on the purpose of 99DOTS
		Good understanding of 99DOTS
		Poor understanding of 99DOTS
	Usefulness for medication adherence and TB treatment	Useful for TB medication adherence
		Helpful for ART adherence
		SMS reminders were helpful
	Not useful for medication adherence and TB treatment	Not useful for TB medication adherence
		Not helpful for ART adherence
		SMS reminders not helpful
	Relationship with providers	Better relationship with providers
		Poorer relationship with providers
	Other life benefits of 99DOTS	Able to work and study
		Easy to pick up HIV and TB medications at same visit
		Saves travel time and money from not having to visit DOT centers
		Other positive statements on usefulness
	Technical problems with phone	Electricity problems
		Battery problems
		Network problems
	Phone access	Has good individual phone access
		No phone available
		Shared phone
		Switched cell phone numbers
		Depending on others to call
		Other problems with phone access

**Effort expectancy**	Poor literacy or steep technology learning curve	Poor literacy or lack of knowledge of English as a barrier to ease of use
		Poor cell phone literacy
	Barriers caused directly by medication	Difficulty in taking TB/ART medication itself
		Medication side effects
		Not calling in spite of side effects
	Issues with envelope	Numbers not visible
		Functioning of envelopes
		Not given medication in envelope
		Problem understanding envelope labeling
	Patient behavior	Fatigue of calling
		No calling of fatigue rerported
		Calling at a different time from medication-taking
		Calling the same number repeatedly or calling a number not in envelope
		Forgetting to call after taking tablets
**Social influences**	Influence of family or community	Increased family involvement and support
		Decreased family involvement
		Stigma or privacy concerns
**Facilitating conditions**	Influence of healthcare worker interaction with patient	Further health system interaction after starting 99DOTS
		No further health system interaction after stargting 99DOTS
	Quality of counseling	Good counseling given about 99DOTS
		Poor counseling given about 99DOTS
	Other health system factors	Barriers caused by distance from health center
