# Supplementary material for: Explaining Differences in the Acceptability of 99DOTS, a Cell Phone–Based Strategy for Monitoring Adherence to Tuberculosis Medications: Qualitative Study of Patients and Health Care Providers
Source: JMIR Mhealth Uhealth. 2020 Jul 31;8(7):e16634. doi: 10.2196/16634 (PMC7428900; doi:10.2196/16634)
Supplement: Multimedia Appendix 2 [file mhealth_v8i7e16634_app2.pdf]

Figure S2. Coding scheme for 99DOTS healthcare provider interviews

Component of the UTAUT framework	Parent nodes	Child nodes
**Performance expectancy**	Barriers to usefulness for healthcare providers	SMS alerts/dashboard unhelpful for management or overwhelming
		Does not save time or lowers work efficiency
	Barriers to usefulness for patients, as perceived by healthcare providers	Reduced patients interactions leading to reduced follow-up
	Facilitators of usefulness for healthcare providers **Text**	SMS alerts or dashboard helpful for patient management
		Saves time or improves work efficiency
	Facilitators of usefulness to patients, as perceived by healthcare providers	Improves quality of care for patients
		Better patient understanding of medications and improved adherence
		Being able to pick up antiretroviral therapy and TB therapy at the same clinic
		Patients perceive improved relationships with providers
		Patient able to work / study without constraints of facility-based DOT
		Interactive voice response perceived as helpful
**Effort expectancy**	Barriers to ease of use for healthcare providers	Difficulties learning 99DOTS
		Difficulty in accessing the SMS/Dashboard alerts
		Issues with dependability of 99DOTS technology
		Does not understand dashboard/SMS alerts
		Does not understand phone reporting process
	Barriers to patient ease of use, as perceived by healthcare providers	Providers think patients find 99DOTS hard to use
		Patient with poor phone access
	Facilitators for ease of use for healthcare providers	Positive statements on ease of use for HCP
		Good provider understanding as evidence of ease of use
	Facilitators for patient ease of use, as perceived by healthcare providers	Positive statements on ease of use for patients
**Social influences**	Influence on provider communication	99DOTS facilitated communication among providers
		99DOTS was barrier to communication among providers
**Facilitating conditions**	Training of HCPs in 99DOTS implementation	No or inadequate training in 99DOTS
		Sufficient training provided in 99DOTS
	Supply chain problems	Supply chain problem for envelopes
		No supply chain problem for envelopes
		Supply chain problem for medications
		No supply chain problem for medications
